# Characteristics of lumbar spondylolysis: L5 versus non-L5

**DOI:** 10.1186/s12891-024-07190-x

**Published:** 2024-01-12

**Authors:** Hisanori Gamada, Masaki Tatsumura, Shun Okuwaki, Reo Asai, Toru Funayama, Masashi Yamazaki

**Affiliations:** 1 Department of Orthopaedic Surgery, Institute of Medicine, University of Tsukuba, 1-1-1 Tennodai, Tsukuba, Ibaraki 305-8575 Japan; 2 Department of Orthopaedic Surgery and Sports Medicine, Tsukuba University Hospital Mito Clinical Education and Training Center, Mito Kyodo General Hospital, Mito, Japan

**Keywords:** Lumbar spondylolysis, Fifth lumbar, Conservative treatment, Characteristics

## Abstract

**Background:**

Fifth lumbar vertebra (L5) spondylolysis has a lower bone union rate than non-L5 spondylolysis, but the reason for this is unknown. This study aimed to evaluate the differences in patient and lesion characteristics between L5 and non-L5 spondylolysis.

**Methods:**

A total of 410 patients with lumbar spondylolysis aged 18 years or younger who were treated conservatively were enrolled. Patients and lesions were divided into L5 and non-L5 (L2–L4) spondylolysis. Factors, including sex, age, presence of spina bifida occulta, stage of the main side lesion, whether the lesion was unilateral or bilateral, presence and stage of the contralateral side lesion and treatment duration, were evaluated at the first visit and compared between the two groups.

**Results:**

A total of 250 patients with 349 lesions were included. The bone union rate of L5 lesions was lower than that of non-L5 lesions (75% vs. 86%, *p* = 0.015). Patients with L5 spondylolysis were more likely to be male (86% vs. 66%) and younger (14.0 vs. 14.6 years) than patients with non-L5 spondylolysis. Lesions of L5 spondylolysis were more likely to be in a progressive stage (28% vs. 15%), less likely to be in a pre-lysis stage (28% vs. 43%) and more likely to be in a contralateral terminal stage (14% vs. 5.3%, *p* = 0.013) compared with lesions of non-L5 spondylolysis.

**Conclusions:**

L5 spondylolysis was characterised by a lower bone union rate, more males, younger age, more progressive stage and more contralateral pseudarthrosis than non-L5 spondylolysis.

## Background

Lumbar spondylolysis is a common fatigue fracture in young athletes, most commonly at the fifth lumbar vertebra (L5) [1, 2]. Early detection by magnetic resonance imaging (MRI) is widespread, and bone union rates for conservative treatment of acute fractures are 77%–100% [3–6].

L5 spondylolysis has a lower bone union rate than non-L5 spondylolysis [7, 8]. Anatomical abnormalities in the lumbosacral spine, such as spina bifida occulta and transitional vertebrae, have been suggested as a reason why lumbar spondylolysis at L5 is common [9–12]. It has also been reported that rotation and extension movements have a significant influence on the development of lumbar spondylolysis [3]. Various studies on the biomechanics of the lumbar spine, including cadaveric, in vivo, and finite element analyses, have reported that the biomechanics of the lumbar spine have different characteristics depending on the level of the spine. The range of motion of the facet joints and the range of motion in rotation, lateral flexion, and flexion–extension differ depending on the vertebral level, with L4/5 and L5/S in particular reported to have a large range of motion in flexion–extension [13–15]. From a biomechanical point of view, the high mechanical stress may be one of the reasons why lumbar spondylolysis occurs more frequently at L5.

However, it is unclear why L5 spondylolysis has a low bone union rate. Although it is clear that lumbar spondylolysis is more likely to occur at L5, both anatomically and biomechanically, the reasons for the low rate of bone union have not been reported.

We focused on the patient characteristics and hypothesised that L5 spondylolysis would have characteristics that differ from those of non-L5 spondylolysis, particularly those previously reported to be unfavourable for bone union. This study aimed to evaluate the differences in patient and lesion characteristics between L5 and non-L5 spondylolysis.

## Methods

This was a retrospective cohort study on 410 patients with lumbar spondylolysis aged 18 years or younger who were treated conservatively in the hospital between April 2014 and March 2022. Inclusion criteria included patients who had completed the hospital’s conservative treatment protocol and who had been assessed for bone union by computed tomography (CT). Exclusion criteria included patients with missing data, patients who dropped out of the protocol during conservative treatment and patients who requested early surgery during conservative treatment. The study methodology was approved by the institutional review board of the hospital. Written informed consent was obtained from all patients and their parents for the publication of this study.

The protocol for conservative treatment was discontinuation of sports, physical education, wearing an extension block brace and athletic rehabilitation. MRI was performed once a month during treatment, and CT was performed when the high signal changes at the fracture site had disappeared to assess bone union. Bone union was defined as the presence of cortical bone continuity in two of the axial, sagittal or coronal planes on CT reconstruction images [4, 8]. Level of lesion and vertebrae counts were assessed by counting the most cephalad vertebra without ribs on CT as L1.

**Fig. 1:**
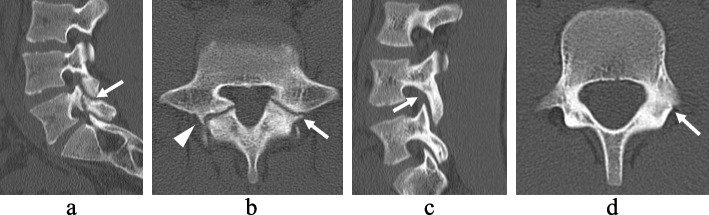
Computed tomography images of L5 and non-L5 spondylolysis. L5 spondylolysis (**a**, **b**). Bilateral lesions are present, the right lesion is a pseudarthrosis with osteosclerosis (arrowhead) and the left lesion is a progressive stage with a clear gap (arrow). Non-L5 spondylolysis (**c**, **d**). Unilateral lesion in L4. Early-stage lesion with hairline (arrow) on the left side only

Patients were divided into groups: patients with L5 spondylolysis and patients with non-L5 (L2–L4) spondylolysis, excluding patients with multilevel spondylolysis. Lesions were divided into groups: lesions of L5 spondylolysis and lesions of non-L5 spondylolysis, including lesions of multi-level spondylolysis (Fig. 1).

Factors, which have been reported to influence bone union, including sex, age and presence of spina bifida occulta (patients characteristics) and stage of the main side lesion, whether the lesion was unilateral or bilateral, presence and stage of the contralateral side lesion and treatment duration (lesion characteristics), were evaluated at the first visit and compared between the two groups [4, 7–10, 16].

If lesions were present on both sides, they were counted as two lesions. Spina bifida occulta, main side lesion stage and contralateral side lesion were assessed by CT. Spina bifida occulta was defined as the absence of continuity between the left and right lamina in at least one location in the lumbosacral spine [12, 16, 17]. Main side lesions were scored in three stages (pre-lysis: only MRI signal change without fracture line on CT; early: hairline on CT; progressive: clear gap on CT) and contralateral side lesions were scored in five stages (none: unilateral; pre-lysis, early, progressive and terminal: pseudoarthrosis without MRI signal change) [1, 4, 7–10, 17, 18]. The treatment period was defined as the time from diagnosis to the disappearance of signal changes on MRI.

### Statistical analysis

Differences in characteristics between the two groups were assessed using the chi-square test and residual analysis for sex, spina bifida occulta and stage as nominal variables and the Mann–Whitney U test for age and treatment duration as continuous variables. All statistical analyses were performed using JMP® 10 (SAS Inc., Cary, NC, USA). The significance level was set at *p* < 0.05.

## Results

**Fig. 2:**
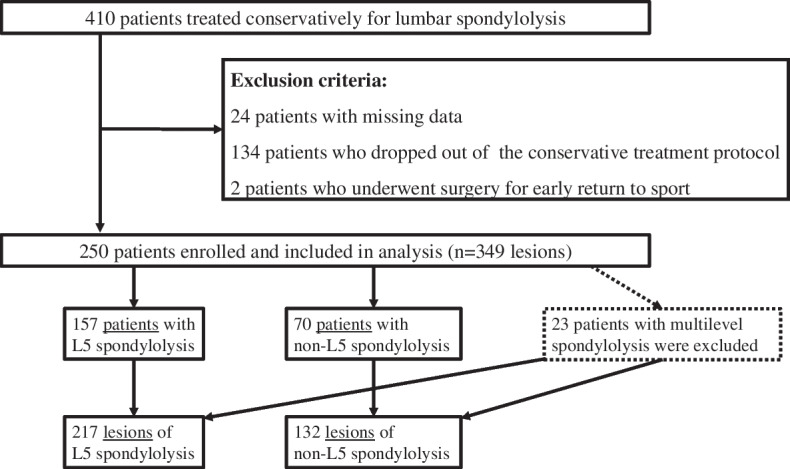
Flowchart of patient inclusion and exclusion criteria

Of the 410 patients, 160 were excluded due to missing data (24 patients), dropping out (134 patients) and undergoing surgery for early return to sport (2 patients). Thus, a total of 250 patients enrolled and included analysis. A total of 227 patients (157 patients with L5 spondylolysis and 70 patients with non-L5 spondylolysis) were included to evaluate the characteristics of the patients, excluding 23 patients with multilevel spondylolysis. A total of 349 lesions (217 lesions of L5 spondylolysis and 132 lesions of non-L5 spondylolysis) were included to evaluate the characteristics of the lesions, including lesions of multilevel spondylolysis (Table 1 and Fig. 2). All 250 patients received conservative treatment for bone union, i.e., lesions in the pre-lysis, early, and progressive stages, and there were no cases of bilateral pseudarthrosis and spondylolisthesis. With regard to the level of lesions and vertebrae, there were no lumbosacral transitional vertebrae in the 250 patients in this study, and all patients had five lumbar vertebrae.

**Table 1:** Patient characteristics

	Patients	Lesions
Total	250	349
Level of lesions		
L5	157	217
Non-L5	70	132
L2	1	5
L3	18	32
L4	51	95
Multi-level	23	n/a
Sex		
Male	200	284
Female	50	65
Mean age (years)	14.3	14.3
Spina bifida occulta		
With	133	179
Without	117	170
Main side stage		
Pre-lysis	n/a	117
Early	n/a	151
Progressive	n/a	81
Unilateral	n/a	128
Bilateral	n/a	221
Contralateral side stage		
None (= unilateral)	n/a	128
Pre-lysis	n/a	49
Early	n/a	76
Progressive	n/a	57
Terminal	n/a	38
Pedicle fracture	n/a	1
Mean conservative treatment duration (days)	n/a	110

### Characteristics of patients with L5 or non-L5 spondylolysis

Patients with L5 spondylolysis were more likely to be male (86% vs. 66%, *p* = 0.0004) and younger (14.0 vs. 14.6 years, *p* = 0.0381) than those with non-L5 spondylolysis. No difference in the presence or absence of spina bifida occulta was observed between the two groups (Table 2).

**Table 2:** Characteristics of patients with L5 or non-L5 spondylolysis

Characteristics	L5	Non-L5	*p*
Total excluding multi-level patients (n)	157	70	
Sex (% [n])			
Male	86% (135)	66% (46)	0.0004^*^
Female	14% (22)	34% (24)	
Mean age (years)	14.0	14.6	0.032^**^
Spina bifida occulta (% [n])			
With	57% (90)	44% (31)	0.069^*^
Without	43% (67)	56% (39)	

^*^Chi-square test; ^**^Mann–Whitney U test

### Characteristics of lesions of L5 or non-L5 spondylolysis

The bone union rates of the total, L5 and non-L5 lesions after conservative treatment were 79% (275/349 lesions), 75% (162/217) and 86% (113/132), respectively. The bone union rate of L5 lesions was lower than that of non-L5 lesions (*p* = 0.015). A significant difference in the distribution of stage was observed between lesions of L5 spondylolysis and lesions of non-L5 spondylolysis, with a more difference in the progressive stage (28% vs. 15%, *p* = 0.0083) and less difference in the pre-lysis stage (28% vs. 43%, *p* = 0.0047) in the residual analysis. Lesions of L5 spondylolysis were more often contralateral terminal stage in the residual analysis (14% vs. 5.3%, *p* = 0.013). No difference in unilateral or bilateral lesions or treatment duration was observed between the two groups (Table 3).

**Table 3:** Characteristics of lesions of L5 or non-L5 spondylolysis

Characteristics	Total	L5	Non-L5	Adjusted residual	*p*
Total	349	217	132		
Bone union after conservative treatment	275	162	113		0.015^*^
Main side stage					0.0025^*^
Pre-lysis	117	60	57	− 2.98	0.0047
Early	151	96	55	0.47	0.36
Progressive	81	61	20	2.78	0.0083
Unilateral	128	79	49		1.00^*^
Bilateral	221	138	83		
Contralateral side stage					0.015^*^
None (= unilateral)	128	79	49	− 0.10	0.40
Pre-lysis	49	24	25	− 2.04	0.050
Early	76	42	34	− 1.38	0.15
Progressive	57	40	17	1.38	0.15
Terminal	38	31	7	2.63	0.013
Pedicle fracture	1	1	0		n/a
Mean conservative treatment duration (days)	110	110	109		0.11^**^

^*^Chi-square test; ^**^Mann–Whitney U test

## Discussion

L5 spondylolysis was characterised by a lower bone union rate, more males, younger age, more progressive stage and more contralateral pseudarthrosis than non-L5 spondylolysis. In a previous study, a multivariable analysis showed that L5 is less favourable for bone union than other levels [7]. It has been suggested that congenital factors related to bone development, such as spina bifida occulta, are involved in the development of L5 spondylolysis, especially in young elementary school children [9, 10].

Anatomically, the lack of local blood flow may contribute to the low bone union rate as the segmental artery is often absent at L5 compared with other lumbar vertebrae [19]. In lumbar spondylolysis, where the fracture line extends from the ventral to the dorsal aspect of the vertebral arch, L5, which has a smaller sagittal diameter of the pedicle than the other lumbar vertebrae, may be more likely to progress to a complete fracture, a progressive stage and the fracture area is smaller, which may be detrimental to bone union [20–22].

Biomechanically, L5 is the lowest of the lumbar vertebrae and forms the lumbosacral transition area, which is the most stressed. It has been reported that the L5/S has a large range of motion and that the contact force on the L5/S facet is greater than that on the L4/5 facet, which has a significant influence on the development of L5 spondylolysis in biomechanics [15, 23, 24]. The high load on the L5/S facet joint may be a factor in the low rate of bone union. In addition, some skeletal studies support the biomechanical theory that sacral tilt and the lumbar lamina and facet joint geometry of the lumbar spine itself are involved in the development of lumbar spondylolysis [25, 26].

Regarding spinal alignment, patients with lumbar spondylolysis have a large sacral slope and an anterior tilt of L5 [27]. Therefore, the fracture line of L5 tends to be more perpendicular to gravity than other lumbar vertebrae, which may result in more shearing forces at the fracture site and be detrimental to bone union. Furthermore, the bone union rate has been reported to be lower in young elementary school children than in middle and high school students, and other progressive and contralateral progressive stages have also been reported to be unfavourable for bone union [4, 8–10, 16, 28]. Additionally, young male children have been reported to have a higher incidence of spina bifida occulta and other anomalies, which may be directly related to the low bone union rate [9–12].

This study is novel in that it has a relatively large sample size of 250 patients compared with previous multivariable analyses and other studies and compares the characteristics of L5 and non-L5 spondylolysis, which have not been reported previously. To the best of our knowledge, this is the first study to address the reasons for the poor bone union rate of L5 spondylolysis. Previous studies have been conducted on L5 spondylolysis and the poor bone union rate of L5 spondylolysis but have not addressed the reason for the poor bone union rates [7, 28]. The study results provide an answer to the clinical question of why conservative outcomes are poor in L5 spondylolysis. Namely, L5 spondylolysis is more common in young boys, who are considered to have more anomalies, mainly spina bifida occulta and more progressive stage and contralateral pseudoarthrosis, which are considered unfavourable for bone union. The characterisation of this L5 spondylolysis may have contributed to the advancement of the treatment system for lumbar spondylolysis. When treating patients with L5 spondylolysis, the ability to diagnose progressive-stage lesions on the main side and terminal-stage lesions on the contralateral side at the initial visit may help patients and physicians make treatment decisions, such as considering the use of low-intensity pulsed ultrasound, which has been reported to improve bone fusion rates, and the indication for early surgery such as internal fixation with screws [29–31].

This study has several limitations. This study aimed to evaluate the causes of low bone union rates in L5 spondylolysis and included only patients who were treated conservatively. Therefore, clear exclusion criteria were established, and the exclusion of 40% of the 410 patients cannot be ruled out as a selection bias. Imaging studies were generally limited to the lumbar spine due to radiation exposure. One other limitation of this study is that it does not adequately evaluate anomalies other than spina bifida occulta, i.e., those that require imaging of the entire spine, such as lumbosacral transitional vertebrae and abnormal vertebral counts. The study was also retrospective, single-centre and single ethnicity, so the generalizability of the results needs to be carefully assessed. However, the fact that the final target population of 250 patients who completed conservative treatment, which is a large number compared with previous reports, is an advantage in terms of generalizability.

## Conclusions

L5 spondylolysis was characterised by a lower bone union rate, more males, younger age, more progressive stage and more contralateral pseudarthrosis than non-L5 spondylolysis. L5 spondylolysis has many factors associated with low bone union rates with conservative treatment and should be treated cautiously.

## Data Availability

The datasets generated during and analysed during the current study are available from the corresponding author on reasonable request.

## Abbreviations

L5: The fifth lumbar vertebrae
MRI: Magnetic resonance imaging
CT: Computed tomography
